# FDG-PET/CT Provides Clues on Bone Marrow Involvement in Follicular Lymphoma and Carries Important Prognostic Information

**DOI:** 10.7150/jca.87523

**Published:** 2023-09-04

**Authors:** Yaoyao Jing, Yafang Chen, Yong Yu, Haifeng Zhao, Hongliang Yang, Bei Sun, Xiaofang Wang

**Affiliations:** 1 Department of Day Ward, Tianjin Medical University Cancer Institute & Hospital, Tianjin, China.; 2 Tianjin Medical University Cancer Institute & Hospital, National Clinical Research Center for Cancer, Tianjin, China.; 3 Tianjin's Clinical Research Center for Cancer, Tianjin, China.; 4 Key Laboratory of Cancer Prevention and Therapy, Tianjin, China.; 5 Department of Hematology, Tianjin Medical University Cancer Institute & Hospital, Tianjin, China.; 6 Department of Hematology, The Second Hospital of Tianjin Medical University, Tianjin, China.

**Keywords:** PET-CT, bone marrow biopsy, follicular lymphoma, prognosis

## Abstract

**Objectives:** To compare the diagnostic efficacy of PET-CT and bone marrow biopsy (BMB) in the detection of bone marrow involvement (BMI) in newly diagnosed patients with follicular lymphoma (FL), as well as their prognostic implications in such patients.

**Methods:** Retrospective analysis was conducted on clinical data from 165 newly diagnosed FL patients. The benefits and drawbacks of PET-CT and BMB in assessing BMI in FL patients were compared and evaluated. Moreover, the prognostic outcomes and factors affecting the survival of FL patients were examined.

**Results:** Among 165 patients, bone marrow involvement (BMI) was diagnosed by PET-CT (PET**^+^**) in 54 cases (32.7%), by bone marrow biopsy (BMB**^+^**) in 50 cases (30.3%), and by either PET**^+^** or BMB**^+^** in 80 cases (48.5%). PET-CT scans upgraded 32 patients (19.4%) to stage IV, including 1 stage I and 4 stage II cases. Four patients were elevated to stage IV by BMB, all of whom had a previous stage III diagnosis. No patients with previous stages I or II were elevated to stage IV by BMB. The median follow-up time was 6.6 years (range,0-11.0 years). The 5-year OS was 86.7%, and the 5-year PFS was 44.8%. Multivariate analysis revealed that BMI by PET-CT was the only independent predictor of PFS reduction. Regarding OS, grade 3a and BMI by PET-CT were independent predictors of decreased survival.

**Conclusion:** PET-CT enables a thorough evaluation of bone marrow involvement in patients with FL, and BMI identified by PET-CT can have substantially implications for patient prognosis. PET-CT obtains vital data for the diagnosis, treatment, and prognosis of FL patients. By contrast, BMB seldom augments crucial data.

## Introduction

Bone marrow involvement (BMI) typically impacts the outcome and management of patients with lymphoma. In those with follicular lymphoma (FL), BMI at initial diagnosis is frequently observed^1^-^3^. This presence can alter the treatment approach for newly diagnosed patients, emphasizing the need for bone marrow evaluation prior to treatment^1^, ^2^, ^4^-^9^.

Bone marrow biopsy (BMB) is a crucial factor in most clinical risk-stratification indices, such as IPI, FLIPI, FLIPI2, PRIMA-PI, IPS^2^, ^8^-^13^. BMB is widely recognized as the gold standard for assessing BMI in lymphoma patients. Nevertheless, some recent studies comparing the utility of PET-CT and BMB in disease evaluation in HL and DLBCL^14^-^18^ have indicated that BMB may not be indispensable for assessing BMI. Instead, PET-CT has shown promising results in assessing the disease status of these two lymphoma types. Likewise, PET-CT's exceptional performance in assessing FL disease is also noteworthy^19^-^21^.

PET-CT can provide valuable information in the pre-treatment phase for FL patients, leading to more precise management^17^. The affinity of 18F-fluorodeoxyglucose (FDG) for lymph nodes has been confirmed in over 95% of FL patients^22^, ^23^. Some studies have also reported 18FDG's effectiveness in evaluating BMI in newly diagnosed FL patients^19^-^22^, ^24^-^30^; however, reported sensitivity varied widely, ranging from 31% to 69%. Additionally, other studies have noted differences in 18FDG affinity between lymph nodes and bone marrow**27**. With this context, this study was conducted to further assess the effectiveness of PET-CT in detecting BMI in newly diagnosed FL patients and its impact on their prognosis.

## Materials and Methods

### Patients

**Figure 1:**
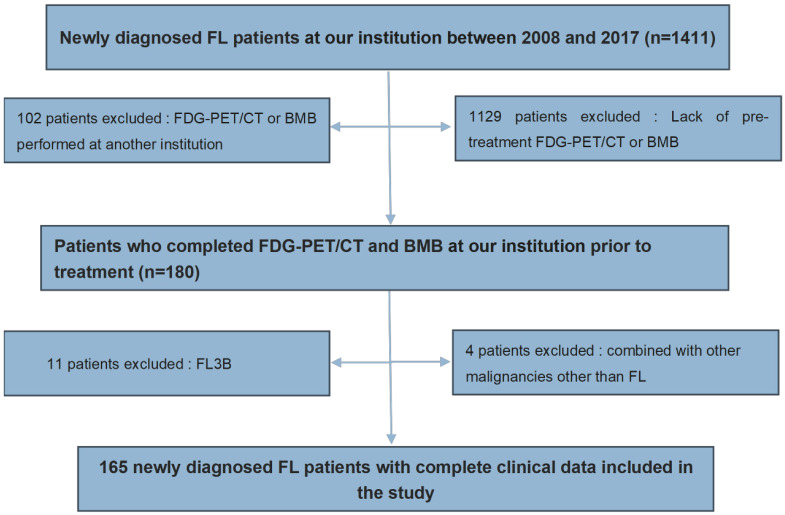
Patient cohort.

A total of 1411 follicular lymphoma patients were newly diagnosed in Tianjin Medical University Cancer Institute & Hospital between January 2008 and December 2017. Our institution's pathologists confirmed the histopathological diagnosis for all patients. Of these individuals, 180 received both PET-CT and BMB evaluations at our institution before treatment. After excluding 11 patients with grade 3b FL and 4 patients combined with malignancies other than FL, a total of 165 newly diagnosed FL patients with grade 1-3a were included in the study (**Figure 1**). All patients included in the study were 18 years of age or older and had no malignancies other than follicular lymphoma. The PET-CT and BMB results were unknown to each other specialist. Studies involving human participants were approved and reviewed by the Medical Ethics Committee of Tianjin Medical University Cancer Institute & Hospital. Informed consent has been obtained from the patients for the study.

The clinical characteristics, pathology, treatment, baseline PET-CT scan results, bone marrow biopsy (BMB) findings, and prognostic survival rates were gathered. The relapse, progression, and survival status of the patients were recorded and analyzed. Studies involving human participants were approved and reviewed by the Medical Ethics Committee of Tianjin Medical University Cancer Institute & Hospital. Progression-free survival (PFS) was determined by calculating the duration from the date of FL diagnosis to the occurrence of disease progression, relapse, or death from any cause. (April 1, 2023). Overall survival (OS) was defined as the interval between the diagnosis of FL and death from any cause or follow-up (April 1, 2023).

### BMB

Unilateral iliac crest trephine biopsy and bone marrow aspirate were performed in all patients with newly diagnosed FL. The results of BMB were confirmed by histopathology rather than flow cytometry or cytology, and the results were reviewed by two experienced hematopathologists.

### PET-CT

**Figure 2:**
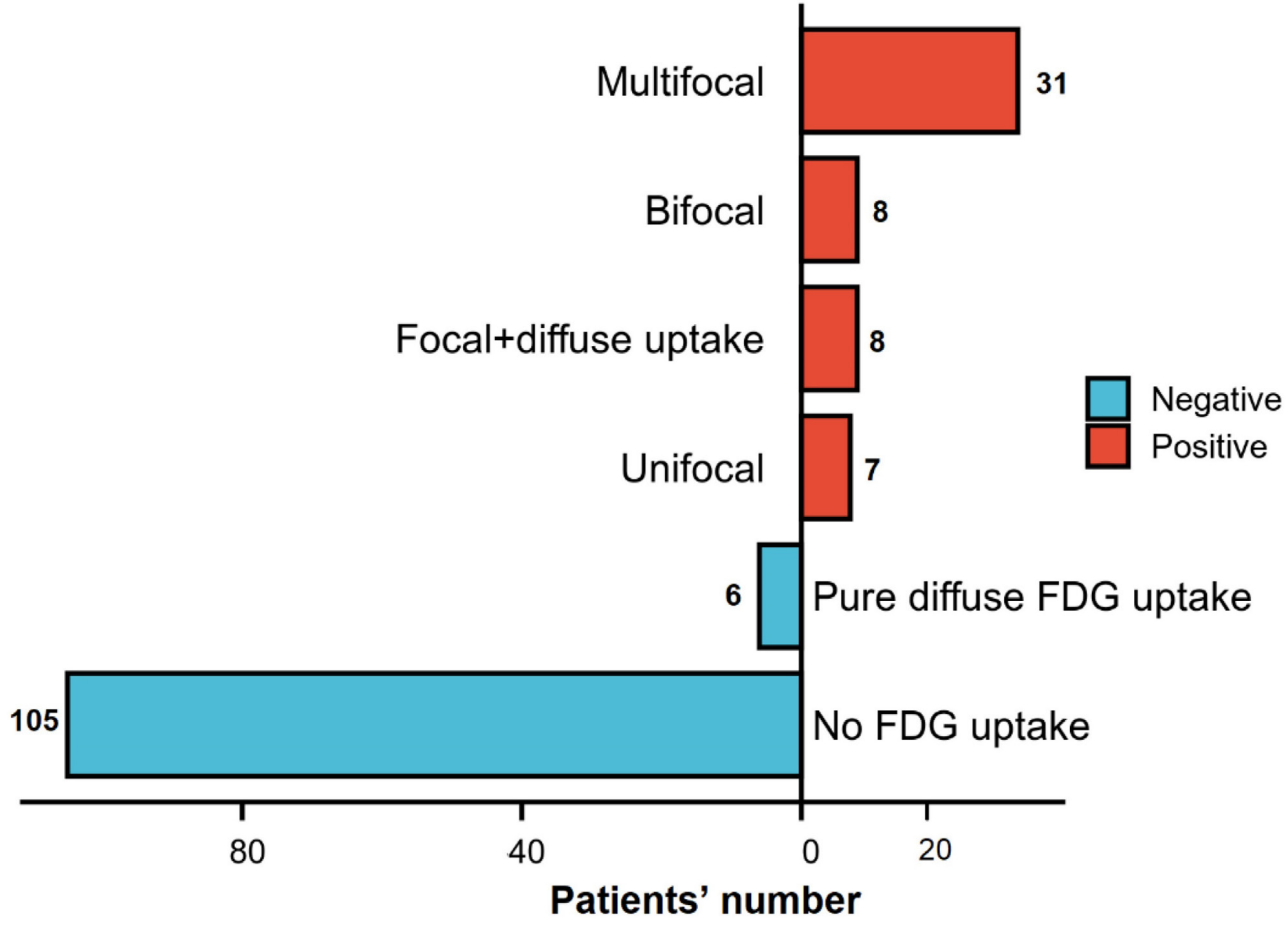
Case distribution of BM status assessed by PET-CT.

All patients were required to fast for a period of at least 6 hours prior to receiving intravenous FDG. During the next 60 to 90 minutes of uptake, patients consumed oral contrast in accordance with institutional protocol. PET images were acquired for 3 minutes per bed position. A positive BMI was determined by the presence of unifocal (single lesion), bifocal (2 lesions), multifocal (≥ 3 lesions), or focal lesions with diffuse uptake. However, purely diffuse FDG uptake was excluded from the diagnosis of BMI (**Figure 2**).

### Statistical Analysis

Categorical data comparisons were assessed using two-sided Fisher exact test or chi-square test. Survival analysis for patients was performed with univariate Cox regression analysis and Kaplan-Meier method, generating forest plots and K-M curves for PFS and OS, respectively. Variables with a significance level of p < 0.2 in the univariate analysis were included in the multivariate analysis to determine independent predictors of PFS and OS. A Cox regression model was utilized for multivariate survival analysis. The statistical significance level was set at p < 0.05. Data analysis was performed using SPSS software (IBM SPSS Statistics 27), GraphPad (GraphPad Software, La Jolla, CA) and R software (R Foundation for Statistical Computing, Vienna, Austria. version 4.2.2).

## Results

### Patient characteristics

A total of 165 newly diagnosed FL patients were included. Clinical characteristics of these patients are presented in **Table 1**. Of these patients, 66 were male and 99 were female, with a gender ratio of 2:3. The median age was 52 years old (25-79). More than half of patients (56.4%) were in Ann Arbor stage IV at the time of diagnosis. Of all patients, 45(27.3%) had high FLIPI scores and 27(16.4%) had high FLIPI-2 scores. The majority of patients had histological grade 1-2 disease (75.8%). At the time of diagnosis, 32 patients (19.4%) had B symptoms. PET-CT imaging identified extranodal diseases in 89 patients (53.9%), as depicted in **Figure 9B**. The primary chemotherapy regimen for most patients was R-CHOP (63.0%). The median follow-up period was 79 months (6-132).

There were 80 individuals with BMI and 85 individuals without BMI (see **Table 3** for details). The 80 FL patients with BMI were divided into two groups: BMB**^+^** and PET**^+^**BMB**^-^**. Clinical characteristics were compared in **Table 2**, which indicated that the clinical characteristics of the two groups were generally similar. However, a higher incidence of histological grade 3a was observed among PET**^+^**BMB**^-^** patients compared to those with BMB**^+^** (p = 0.021).

### Performance of PET-CT and BMB in diagnosing BMI

A total of 60 patients (36.4%) had abnormal FDG uptake on PET-CT, of which 31 (18.8%) had multifocal BM lesions, 8 (4.8%) had bifocal BM lesions, 7 (4.2%) had unifocal BM lesions, 8 (4.8%) had focal lesions with diffuse FDG uptake, and 6 (3.6%) showed purely diffuse FDG uptake. The remaining 105 patients did not exhibit any increased FDG uptake on PET-CT. According to the aforementioned definition, BMI was identified by PET in 54 patients (32.7%) (**Figure 2**).

Out of the 165 patients, 54 (32.7%) were diagnosed with BMI through PET-CT scans, and among them, 30 were found to have a negative BMB. Additionally, 50 patients (30.3%) received a positive BMI diagnosis through BMB, with 26 of them having a negative PET result for BMI. There were 24 patients (14.5%) who were diagnosed with BMI by both PET and BMB, 80 patients (48.5%) by either PET or BMB. BMI was not detected in 85 patients (51.5%). Of the 165 patients, 30 PET-positive BMI was missed by BMB and 26 PET failed to identify BMB-positive BMI. As shown in **Table 3**.

Focusing on the performance of PET-CT and BMB in the diagnosis of BMI, taking "combined BMB and PET-CT" as gold standard for detecting BMI, the sensitivity of PET-CT was 67.5% (95% CI: 56.0-77.3), accuracy was 84.2 (95% CI: 77.9-89.1), and negative predictive value (NPV) was 76.6 (95% CI: 67.4-83.9). For BMB, the sensitivity was 62.5 (95% CI: 50.9-72.9), accuracy was 81.8 (95% CI: 75.2-87.0), and NPV was 73.9 (95% CI: 64.7-81.5) (**Table 4**).

### Performance of PET-CT and BMB in staging

**Figure 3:**
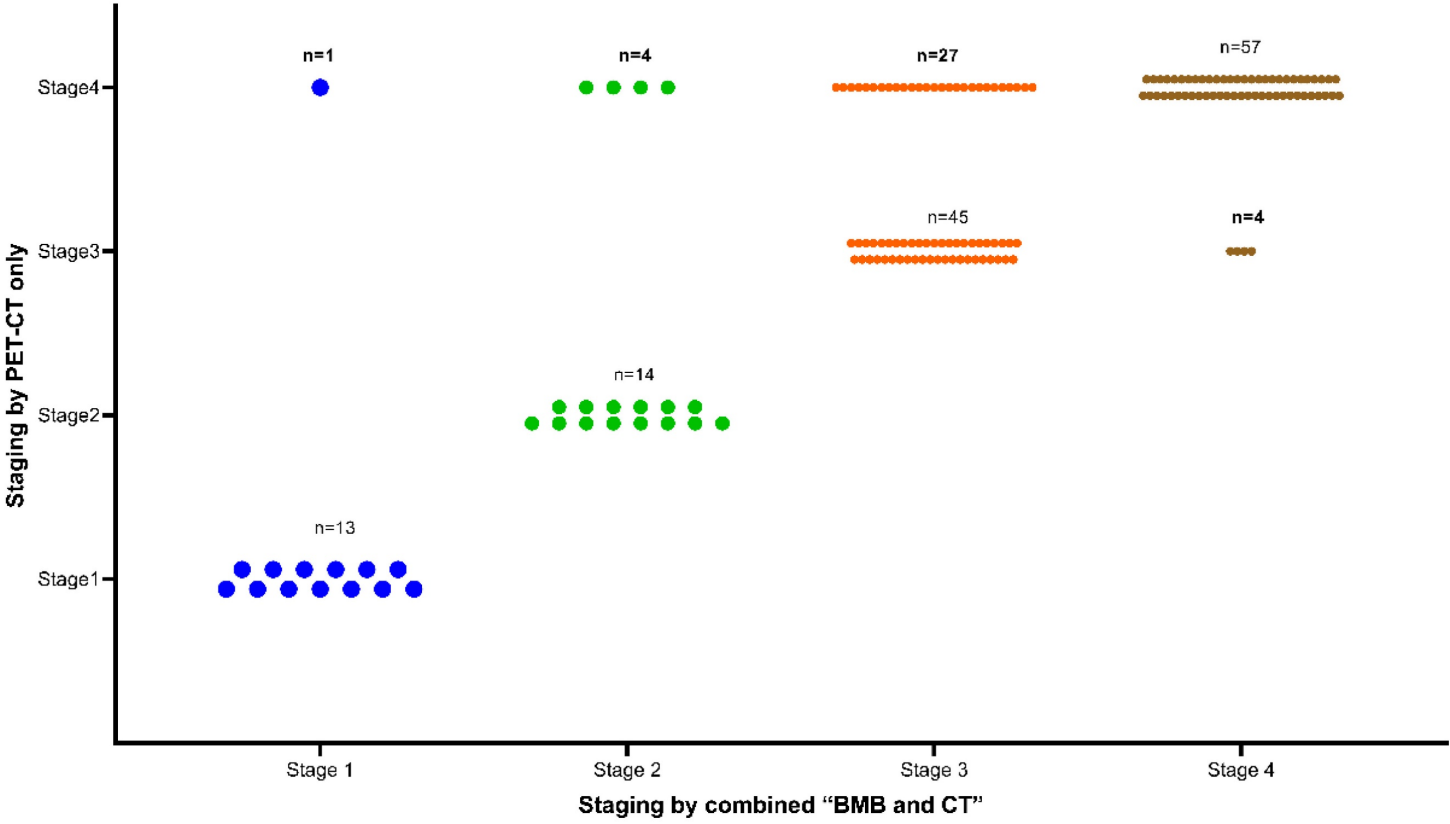
Clinical stage using “combined BMB and CT” or “PET-CT only” as reference standard (n=165).

Overall, 93 (56.4%) patients were diagnosed with stage IV disease. As shown in Figure 3, due to positive PET-CT findings, 32 patients were upgraded to stage IV. Of these 32 patients, 27 were previously classified as stage III. Interestingly, 5 BMB**^-^** patients with previous early stage (1 stage I, 4 stage II) were reclassified as stage IV due to the addition of PET-CT findings. The inclusion of PET-CT reclassified these 5 patients from an early to a late stage, consequently impacting the selection of their treatment course. Conversely, BMB**^+^** findings led to 4 patients being upgraded to stage IV from their previous stage III classification. Notably, none of the patients with a previous early stage (stage I or II) had an elevated stage due to the addition of BMB (**Figure 3**).

### Prognostic significance of PET-CT and BMB

There were 129 survivors (78.2%) at the data cutoff, of whom 128 (77.6%) had been followed for more than 5 years. A total of 101 patients (61.2%) had disease progression or recurrence at the data cutoff. The median follow-up time for all patients was 6.6 years (range,0-11.0 years). The estimated 5-year OS was 86.7%, and the 5-year PFS was 44.8%.

**Figure 4:**
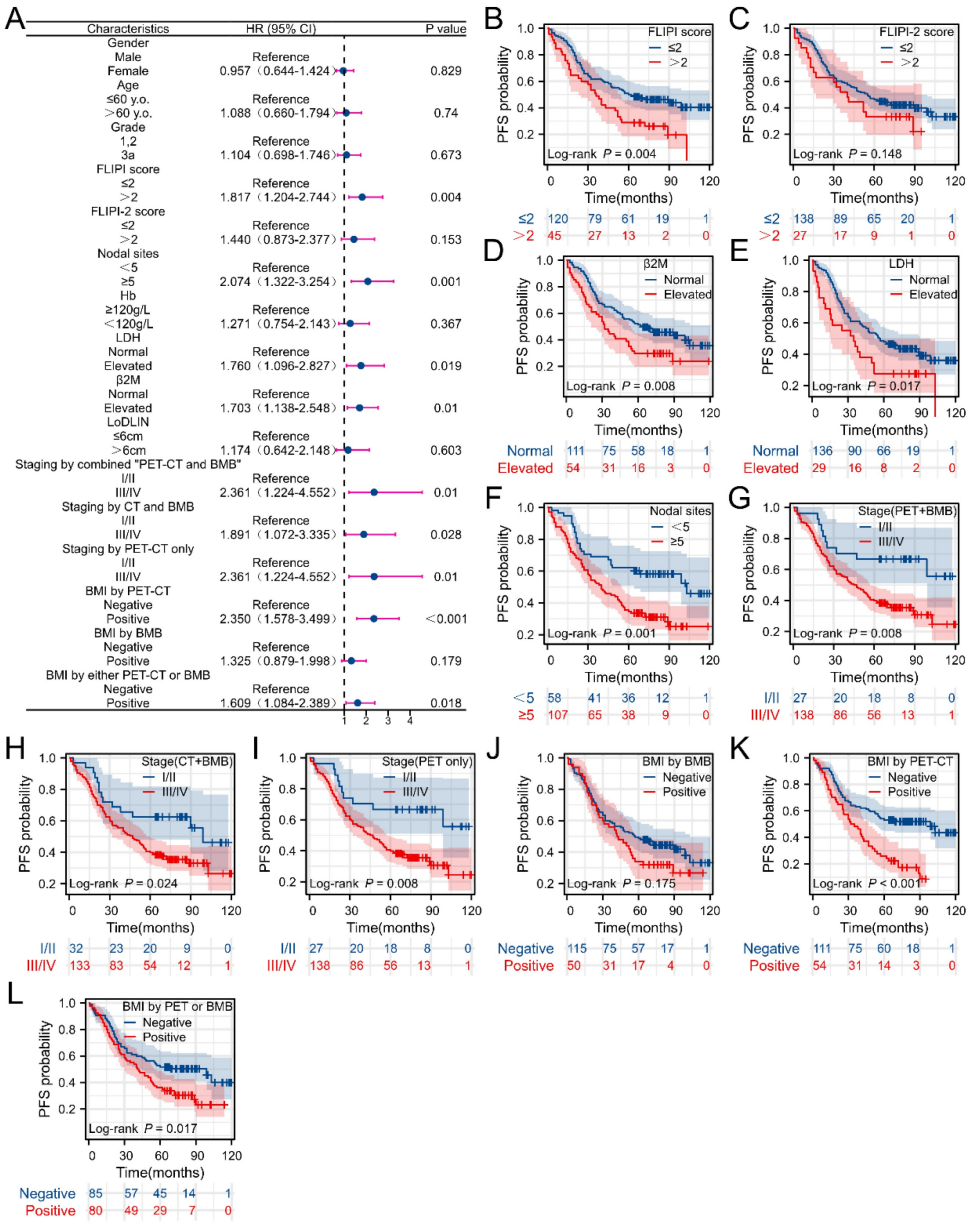
Correlations between clinical characteristics and progression-free survival (PFS) in FL patients. (A) Forest plot of results from the univariate survival analysis for PFS. (B-L) Kaplan-Meier survival curves showed correlations between clinical characteristics and PFS.

**Figure 5:**
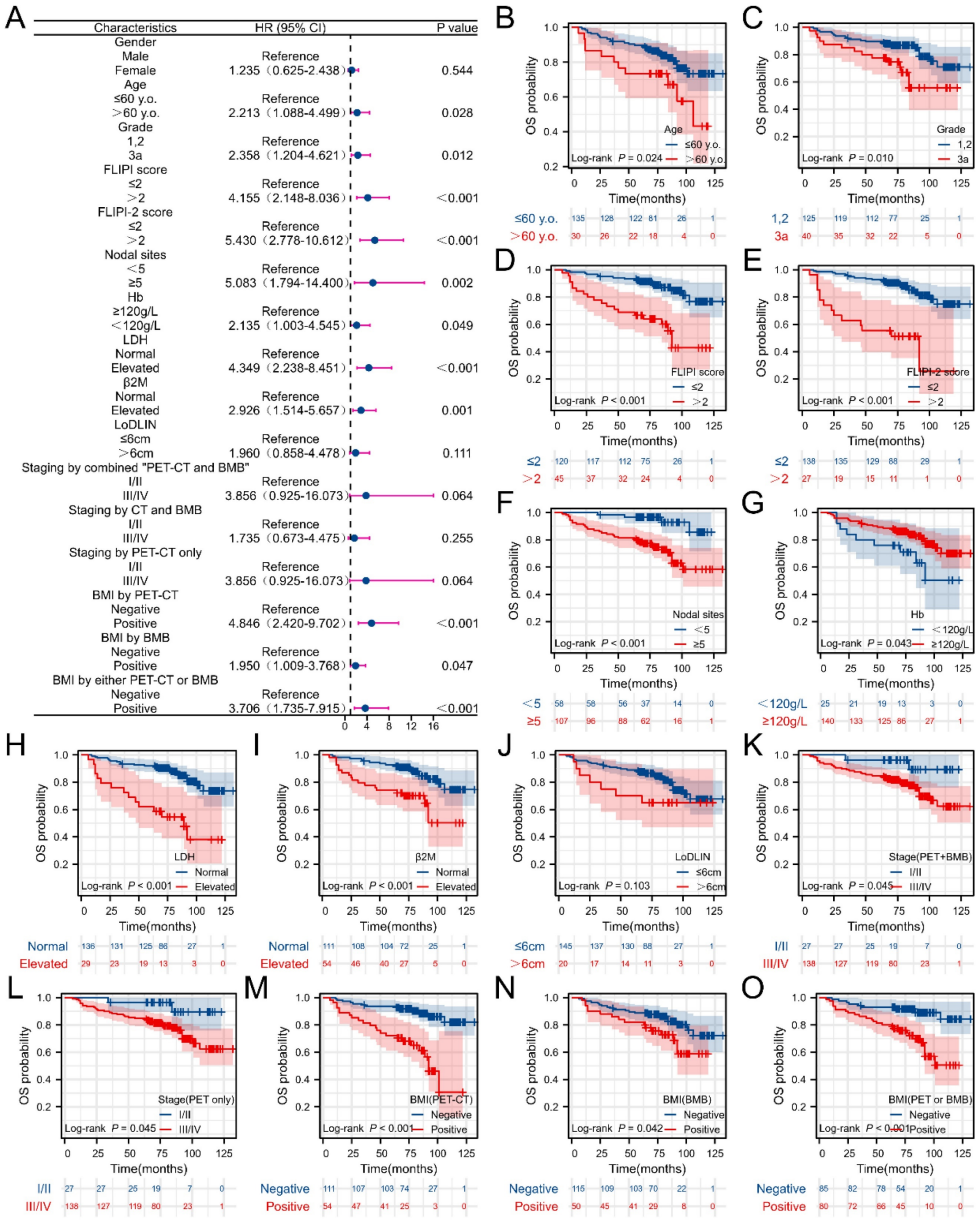
Correlations between clinical characteristics and overall survival (OS) in FL patients. (A) Forest plot of results from the univariate survival analysis for OS. (B-O) Kaplan-Meier survival curves showed correlations between clinical characteristics and OS.

Univariate analysis revealed the correlation between clinical parameters and PFS as well as OS in FL patients, as depicted in **Figures 4 and 5**. Univariate analysis of PFS indicated that FLIPI score > 2, elevated β2M levels, increased LDH levels, ≥ 5 nodal sites, stage III/IV (determined by PET+BMB, CT+BMB or PET alone), BMI (by PET or “PET or BMB”) were significantly associated with reduced PFS (**Figure 4B, D-I, K-L**). However, BMI by BMB was not associated with PFS (**Figure 4A**). For OS, age > 60 years, grade 3a, FLIPI score > 2, FLIPI-2 score > 2, ≥ 5 nodal sites, Hb < 120 g/L, elevated LDH, elevated β2M, stage III/IV (PET+BMB or PET alone), BMI (by PET, BMB or “PET or BMB”) were significantly associated with decreased OS (**Figure 5 B-I, K-O**). It should be noted that to achieve greater precision and comprehensiveness in our statistical findings, we employed distinct statistical methods for the forest plots and K-M curves depicted in **Figures 4 and 5**. Specifically, univariate cox analysis was applied for the forest plots, while log-rank testing was utilized for the K-M curves. Therefore, the resultant P values are marginally dissimilar between the two approaches.

**Figure 6:**
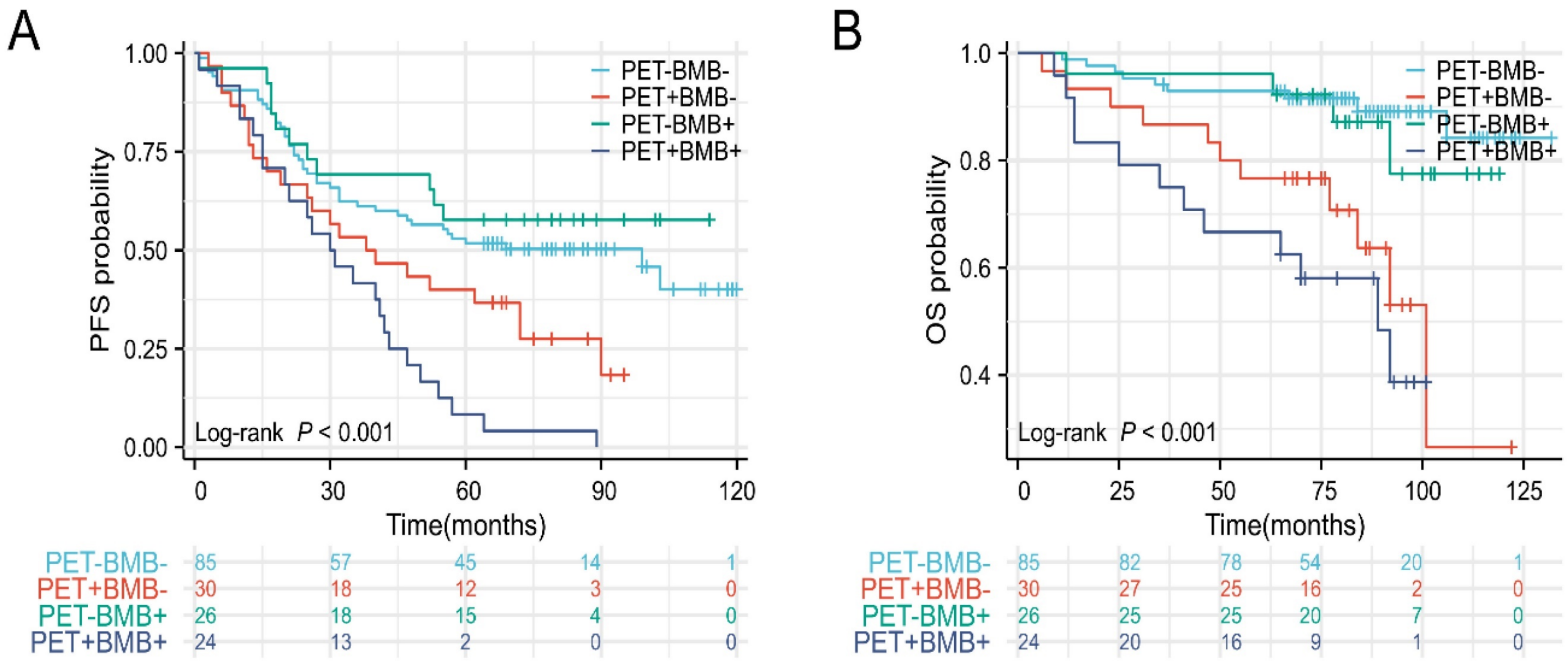
The clinical efficacy comparison of PET-CT and BMB in FL patients. (A) Kaplan-Meier survival curves of PFS. (B) Kaplan-Meier survival curves of OS.

The clinical efficacy comparison of PET-CT and BMB in FL patients was shown in **Figure 6**. As shown in **Figure 6A**, patients in the PET**^+^** subgroup, regardless of BMB status, had the worst PFS (p< 0.001), while those in the PET**^-^**BMB**^+^** subgroup had the longest PFS. Generally, patients with BMI have worse prognoses (PFS and OS) compared to those without (see **Supplemental Figure 1**). However, in the PET**^-^**BMB**^+^** subgroup, BMB positivity did not alter the fact that this subgroup had the best PFS. Consequently, PET**^-^** appears to be a protective factor for PFS in FL patients. In addition, patients in the PET**^+^** subgroup, BMB**^+^** or BMB**^-^**, had the shortest OS (**Figure 6B**, p< 0.001). These results suggest that PET-CT is a better predictor of clinical outcome in FL than BMB.

**Figure 7:**
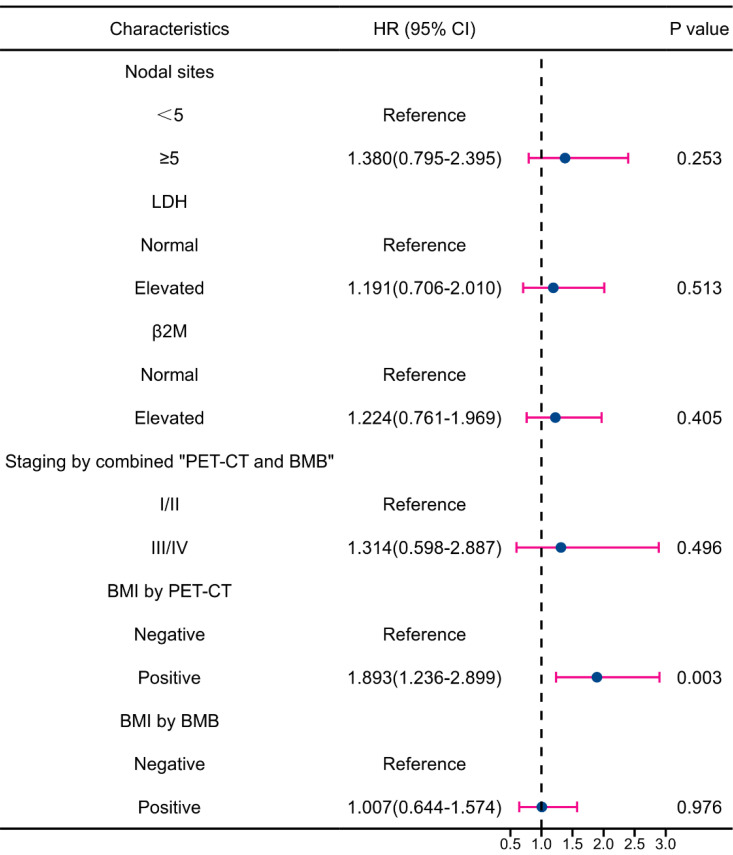
Forest plot of results from the multivariate survival analysis for progression-free survival (PFS).

**Figure 8:**
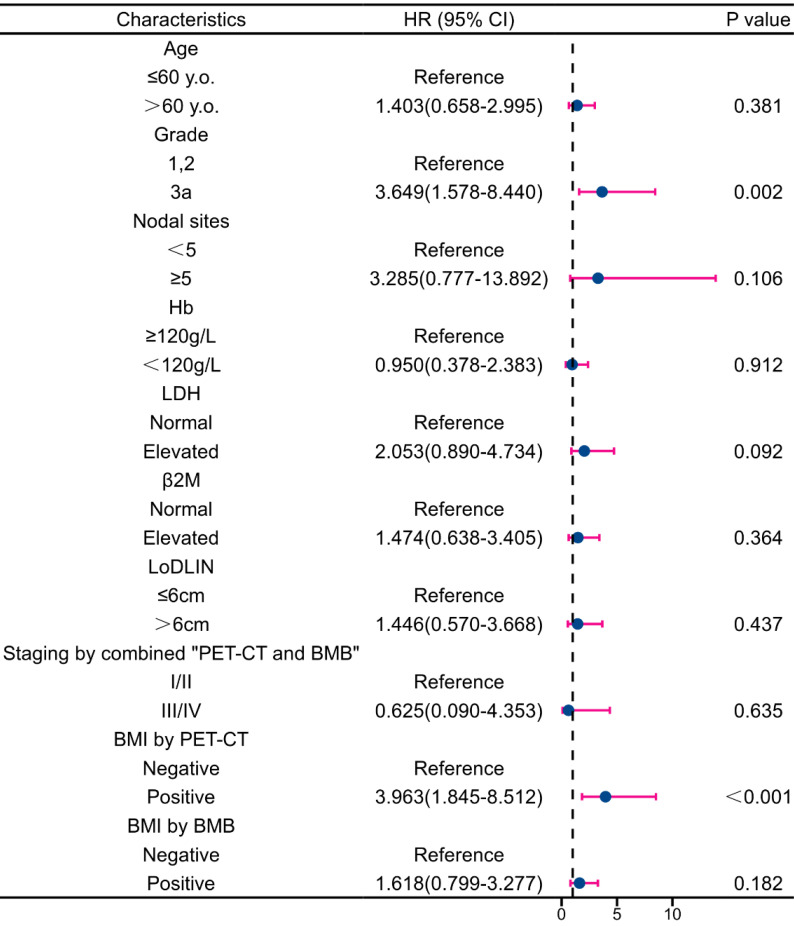
Forest plot of results from the multivariate survival analysis for overall survival (OS).

Multivariate analysis revealed that BMI by PET-CT was the only independent predictor of PFS reduction (**Figure 7**). Regarding OS, grade 3a and BMI by PET-CT were independent predictors of decreased survival (**Figure 8**). Notably, FLIPI score, FLIPI-2 score, stage by CT+BMB, stage by PET alone, and BMI by PET or BMB were excluded from the multivariable analysis of PFS due to their high correlation with the analyzed indicators, which may result in collinearity bias. Similarly, FLIPI score, FLIPI-2 score, stage by PET-CT alone, and BMI by PET or BMB were omitted from the multivariable analysis of OS for the same reason.

### Additional positive PET-CT findings

**Figure 9:**
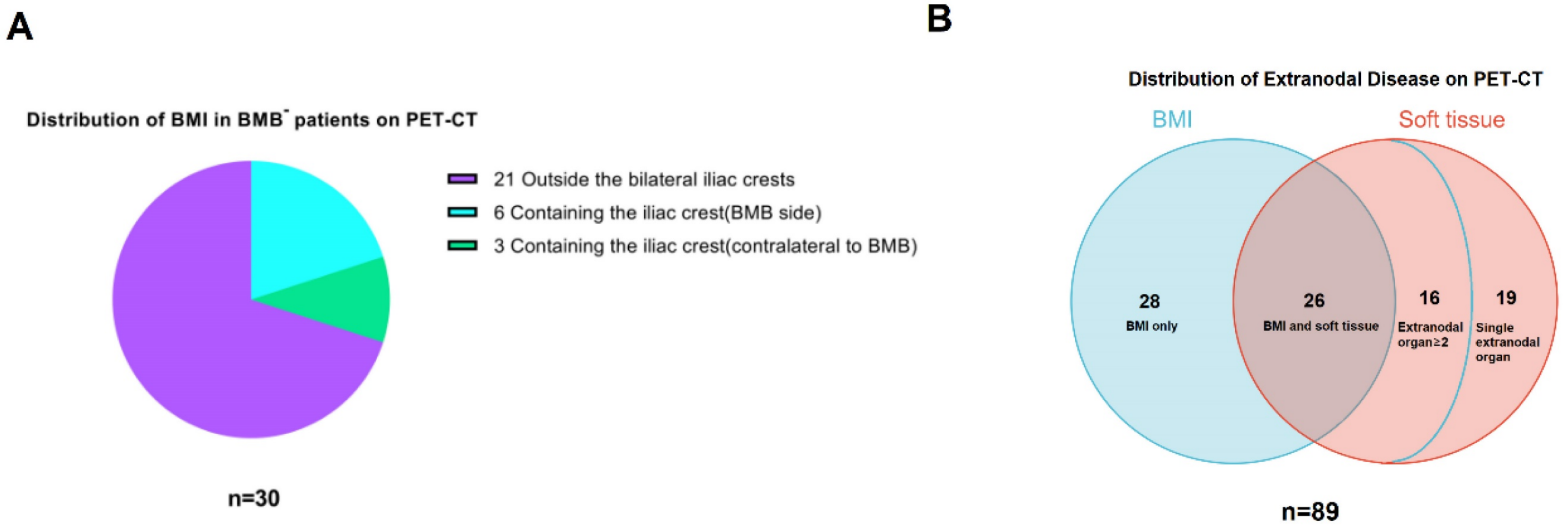
Positive PET-CT findings. (A) Distribution of BMI in BMB^-^ PET^+^ patients(n=30). (B) Distribution of extranodal disease on PET-CT in stage IV patients(n=89).

Of the 54 PET**^+^** patients, only 24 were BMB**^+^** and 30 were BMB**^-^**. This suggests that BMI may be missed by BMB in this group of patients. Further analysis indicated that among the 30 BMB**^-^** PET**^+^** patients, most (70.0% (21/30)) had BMI in sites outside the iliac crests, such as vertebrae and humerus. Only 9(30% (9/30)) patients had iliac crest(s) involvement. It should be highlighted that in 3 of these 9 patients, the BMI was unilateral and on the opposite side of the iliac crest from where the BMB was performed. Thus, BMB failed to detect BMI in these cases. Moreover, it's worth noting that for the remaining 6 patients, whose positive PET-CT results included the iliac crest on the BMB side, it is highly unlikely that the PET-CT results were false positive due to BMB. This is supported by the fact that the PET-CT scans of these 4 patients were performed prior to BMB, and in the case of the remaining 2 patients, the interval between BMB and PET-CT was over a week. See **Figure 9A** for details.

A total of 89 patients were diagnosed with stage IV disease by PET-CT. The distribution of extranodal disease is shown in **Figure 9B**. Of these patients, 54 (60.67%) had BMI and 61 (68.54%) had soft tissue involvement. Twenty-eight patients had BMI only, 35 had soft tissue involvement only, and 26 had both bone marrow and soft tissue involvement. In total, 42 patients (26 + 16) had two or more extranodal organs involved, accounting for 47.19% (42/89) of the cases.

## Discussions

PET-CT plays a crucial role in staging and guiding treatment for patients with malignant lymphoma. For diffuse large B cell lymphoma and Hodgkin lymphoma, PET-CT can almost replace BMB for BMI detection^16^. However, for FL patients, BMB is still a routine pre-treatment test^31^. Against this background, our findings suggest that PET^+^ patients have a comparable FL tumor burden to BMB^+^ patients. Moreover, BMB may not be necessary for FL patients who have already undergone PET-CT, which is supported by the following conclusions: 1) BMB seems unnecessary for FL patients considered to have early -stage disease by PET-CT, as none of them were assigned to a high clinical risk group due to positive BMB results. 2) Omitting BMB for 50 stage IV BMB^+^ patients and only undergoing PET-CT did not result in any misassignment to a lower clinical risk group, with only 4 patients being reassigned to stage III. Furthermore, our research shows that BMI by PET-CT independently affects outcomes (PFS and OS) in FL patients, while BMI by BMB has no independent effect on patient survival, further confirming the suspected redundancy of BMB.

In fact, for patients with advanced-stage disease identified by BMB, there is usually evidence of other advanced illnesses^32^, ^33^. In our study, 96% of BMB-positive patients revealed simultaneous involvement in the spleen, liver, lungs, adnexa, and other areas. This shows that PET-CT can offer the same guidance as BMB for this patient group. Biopsies can potentially induce discomfort, including bleeding, anxiety, and pain^34^, ^35^. Therefore, careful consideration is needed when determining the necessity of performing a BMB.

Accurate staging is crucial for the treatment of patients with stage I/II FL who may receive radiotherapy alone^36^. Misdiagnosis of a spreading disease as a localized one may result in ineffective therapy. PET-CT can lead to staging changes in 10% to 30% of lymphoma patients, more often elevating the stage^16^. Improving staging accuracy reduces overtreatment or undertreatment of patients^16^. Several reports have clarified the use of PET-CT in FL patients. St-Pierreet et al.^20^, ^21^ reported a series of studies on PET-CT and FL. Their work indicated that the addition of PET-CT could lead to an elevated stage in 16% of FL patients, which was also reported by Luminari et al.**22** at 7.5%, Nakajima et al.**19** at 9.2%, and Le Dortz et al.**24** at 18%. In our study, PET-CT led to a stage change in 21.8% of FL patients, with 19.4% assigned to a higher clinical stage. Specifically, PET-CT elevated clinical stage (from stage I/II to stage IV) in 5 patients, enabling us to devise an accurate treatment plan. Nakajima et al.**19** reported that 6 patients who were classified as early stages by PET-CT were reassigned to Stage IV by BMB, which altered their treatment plan. However, in our study, BMB upstaged only 4 patients (considered stage III by PET-CT) to stage IV, yet it did not affect their clinical risk grouping.

A unilateral iliac crest BMB only examines a small portion of the total bone marrow, which is supported by the fact that only unilateral involvement can be found when bilateral BMB are performed^37^. Study^19^ have revealed that a majority of BMI sites are located beyond the iliac crests, suggesting that BMB may fail to identify BMI that is not located in the iliac crests. This is consistent with the results of our study. We analyzed BMI distribution in 30 PET^+^BMB^-^ patients and found that 24 patients (80%) had BMI omission due to lack of iliac crest involvement on the BMB side. Performing directional biopsies in patients with BMI on PET-CT is impractical, therefore, we observed patients' post-treatment responses. We found that most baseline PET-positive BMI lesions showed a substantial reduction or completely disappeared.

Previous studies^22^, ^26^, ^28^-^30^ have reported the predictive impact of PET-CT on BMI in FL patients, with sensitivity ranging from 31% to 68%, which may be attributed to differences in reference standards used in various studies. In the study conducted by Nakajima et al.^19^, PET-CT had a diagnostic performance of 69% sensitivity and 87% accuracy. Similar to their findings, the current study reported a sensitivity of 67.5% and accuracy of 84.2% for PET-CT in predicting BMI. However, it should be noted that, comparative diagnostic studies assessing BMB versus PET-CT in this context are inherently biased due to the absence of an objective reference standard^15^, ^38^. Thus, the predictive capacity of each method may serve as a more reliable indicator of clinical usefulness.

Our analysis revealed that detection of BMI by PET-CT was an independent prognostic factor for both PFS and OS in FL patients, while BMI by BMB did not independently affect prognosis. Further subgroup analysis of BMI patients indicated a higher prevalence of PET^+^BMB^-^ patients than BMB^+^ patients in histological grade 3a, suggestive of their more aggressive disease. Consistent with previous studies^1^-^3^, FL patients commonly present with BMI at diagnosis (40-70%) and rare B symptoms. In this study, approximately 48.5% of patients presented with BMI and 19.4% with B symptoms at initial diagnosis.

Several previous studies 24-26, 29, 30, 39, 40 have investigated the role of PET-CT in determining BMI in patients with FL, however, most of them are small sample studies before 2020. More recently, two studies^19^, ^41^ with larger sample sizes have been conducted. Nakajima et al.'s^19^ study, which included 261 patients, concluded that BMI by PET-CT was the only independent predictor of PFS in FL patients, while a high FLIPI score and BMI by PET-CT predicted lower OS. However, collinearity bias may have affected their results due to the presence of duplicated factors in multivariate analysis. In our study, we deconstructed FLIPI and FLIPI-2 and excluded variables containing duplicate factors in multivariate analysis to eliminate this bias. Our study's conclusion supports the use of BMI by PET-CT in independently predicting PFS in FL patients, while BMI by PET-CT and grade 3A can independently impact OS of FL patients. Although our conclusion is similar to that of Nakajima et al.^19^, BMI by PET-CT was still an independent predictor of outcome in FL patients after controlling for bias, which makes this conclusion more conclusive. Rodenas-Quinonero I et al.^41^ conducted another larger study involving 299 patients and concluded that BMI determined by “PET-CT or BMB” provided independent prognostic guidance for OS in FL patients. However, this contradicts our conclusion of omitting BMB due to its lack of prognostic significance and minimal impact on clinical risk grouping and treatment choice. Moreover, it is worth mentioning that the median follow-up duration of the two prior studies was 5.9 years and 4.75 years (57 months), respectively. In comparison, the present study's median follow-up length extended beyond 6.5 years, enabling us to diligently monitor patient endpoints and yield more precise outcomes.

However, the study still has some limitations. Firstly, it is a retrospective study, and patients may experience recall bias during follow-up. Secondly, it is a single-center study with a limited sample size. Thirdly, directional biopsy could not be performed on the PET+ patients due to technical constraints. Fourthly, the issue of diffuse FDG uptake as a manifestation of BMI is controversial. Some reports suggest a high false-positive rate^29^ of diffuse FDG uptake, while another small series confirm its association with BMI^42^. Our study did not consider simple diffuse FDG as BMI, requiring further investigation. In conclusion, we anticipate the development of prospective, multi-center, large-sample studies to provide more accurate diagnostic and therapeutic guidance for FL patients.

In summary, PET-CT can enhance the accuracy of diagnosis and treatment for patients with FL. Additionally, BMI positivity by PET-CT can serve as an independent prognostic tool for FL patients, highlighting the crucial role of PET-CT in the pretreatment evaluation of newly diagnosed FL patients.

## Supplementary Material

Supplementary figure.

## Figures and Tables

**Table 1:** Patient characteristics(n=165)

Characteristic	No. of patients	Percentage
Gender		
Male	66	40%
Female	99	60%
Age, y.o.		
Median	52	
Range	25-79	
Ann Arbor Stage at diagnosis		
I	14	8.5%
II	13	7.9%
III	45	27.3%
IV	93	56.4%
FLIPI		
Low	52	31.5%
Intermediate	68	41.2%
High	45	27.3%
FLIPI-2		
Low	104	63.0%
Intermediate	34	20.6%
High	27	16.4%
Grade		
1-2	125	75.8%
3a	40	24.2%
B symptoms at diagnosis	32	19.4%
Extranodal diseases on PET-CT	89	53.9%
Initial treatment		
RCHOP	104	63.0%
CHOP	25	15.2%
CHOPE	8	4.8%
FC	6	3.6%
FA	3	1.8%
REPOCH	3	1.8%
RCOP	2	1.2%
R	2	1.2%
RFC	2	1.2%
RFA	2	1.2%
RCOPE	1	0.6%
RCVP	1	0.6%
Observation	3	1.8%
Radiation	3	1.8%
Follow-up, median (range), mo	79 (6-132)	

Abbreviations: y.o, years old; R, rituximab; CHOP, cyclophosphamide, doxorubicin, vincristine, and prednisone; CHOPE, CHOP, etoposide; FC, fludarabine, cyclophosphamide FA, fludarabine, doxorubicin; REPOCH, R, etoposide, prednisone, vincristine, cyclophosphamide, doxorubicin; RCOP, R, cyclophosphamide, vincristine, and prednisone; RCOPE, RCOP, etoposide; RCVP,R, cyclophosphamide, vincristine, prednisone.

**Table 2:** Baseline features of patients with positive BMB Versus patients with BMI by PET-CT and negative BMB(n=80).

Characteristic	Total(n=80)	BMB^+^(n=50), n(%)	PET^+^BMB^-^(n=30), n(%)	p
Gender				0.767
Male	31	20(40.0%)	11(36.7%)	
Female	49	30(60.0%)	19(63.3%)	
Age, y.o.				0.160
>60	15	7(14.0%)	8(26.7%)	
≤60	65	43(86.0%)	22(73.3%)	
Median		51	53	
Range		25-76	26-78	
Grade				**0.021**
1,2	64	44(88.0%)	20(66.7%)	
3a	16	6(12.0%)	10(33.3%)	
B symptoms				0.249
Yes	19	14(28.0%)	5(16.7%)	
No	61	36(72.0%)	25(83.3%)	
FLIPI				0.283
Low	11	7(14.0%)	4(13.3%)	
Intermediate	40	28(56.0%)	12(40.0%)	
High	29	15(30.0%)	14(46.7%)	
FLIPI-2				0.958
Low	33	20(40.0%)	13(43.3%)	
Intermediate	22	14(28.0%)	8(26.7%)	
High	25	16(32.0%)	9(30.0%)	
Nodal sites				1.000
<5	13	8(16.0%)	5(26.7%)	
≥5	67	42(84.0%)	25(83.3%)	
Hb				1.000
<120g/L	13	8(16.0%)	5(16.7%)	
≥120g/L	67	42(84.0%)	25(83.3%)	
LDH				0.309
Normal	61	40(80.0%)	21(70.0%)	
Elevated	19	10(20.0%)	9(30.0%)	
β2M				0.133
Normal	42	23(46.0%)	19(63.3%)	
Elevated	38	27(54.0%)	11(36.7%)	
LoDLIN				0.520
≤6cm	68	41(82.0%)	27(90.0%)	
>6cm	12	9(18.0%)	3(10.0%)	
Treatment				0.481
Contains R	67	43(86.0%)	24(80.0%)	
Does not contain R	13	7(14.0%)	6(20.0%)	

Abbreviations: y.o, years old; Hb, hemoglobin; LDH, lactate dehydrogenase; β2M, β2-microglobulin; LoDLIN, longest diameter of the largest involved node; R, rituximab.

**Table 3:** Comparison of diagnostic performance between PET-CT and BMB in detecting BMI.

	PET+	PET-	Total
**BM+**	24	26	50
**BM-**	30	85	115
**Total**	54	111	165

**Table 4:** Detection of BMI.

	Combined “BMB and PET-CT” as a reference standard for BMI	
	BMI by PET-CT [Percentage (95%CI)]	BMI by BMB [Percentage (95%CI)]
Sensitivity	67.5(56.0-77.3)	62.5(50.9-72.9)
Accuracy	84.2(77.9-89.1)	81.8(75.2-87.0)
NPV	76.6(67.4-83.9)	73.9(64.7-81.5)

Abbreviations: NPV: negative predictive value.

## Abbreviations

BMB: bone marrow biopsy
BMI: bone marrow involvement
FL: follicular lymphoma
FLIPI: Follicular Lymphoma International Prognosis Index
β2M: β2-microglobulin
LDH: lactate dehydrogenase
PFS: progression-free survival
OS: overall survival
