# Supplementary material for: FDG-PET/CT Provides Clues on Bone Marrow Involvement in Follicular Lymphoma and Carries Important Prognostic Information
Source: J Cancer. 2023 Sep 4;14(14):2726–38. doi: 10.7150/jca.87523 (PMC10539392; doi:10.7150/jca.87523)
Supplement: Supplementary file 1 — Supplementary figure. [file jcav14p2726s1.pdf]

Supplemental Figure 1：Embedded histogram showing 5-year PFS and 5-year OS of 165 FL patients. (A) Comparison of 5-year PFS in "BMI by either PET-CT or BMB" (n=80) and "No BMI" (n=85) patients. (B) Comparison of 5-year OS in "BMI by either PET-CT or BMB" and "No BMI" patients.

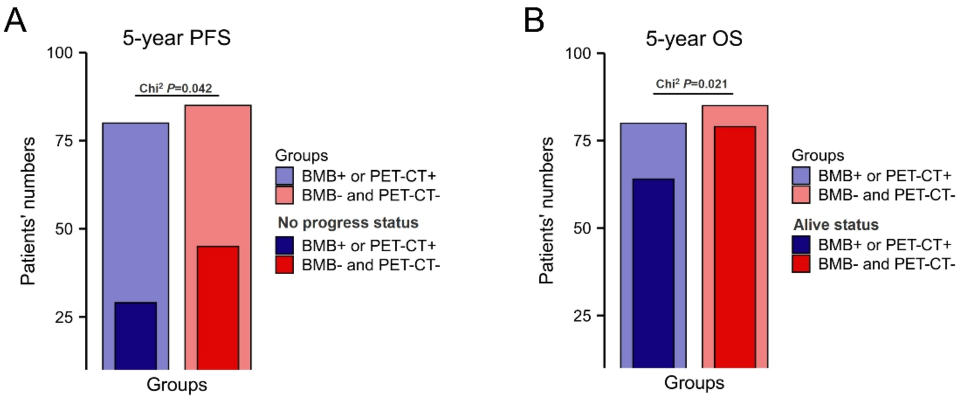

Notes:

Figure 1A: The 5-year progression-free survival (PFS) rates in the BMI and non-BMI groups were 36.3% (29/80) and 52.9% (45/85), respectively, showing a statistically significant difference (p = 0.042).

Figure 1B: The 5-year overall survival (OS) rates in the BMI and non-BMI groups were 80.0% (64/80) and 92.9% (79/85), respectively, showing a statistically significant difference (p = 0.021).
